# Macular Changes in a Mucopolysaccharidosis Type I Patient with Earlier Systemic Therapies

**DOI:** 10.1155/2021/8866837

**Published:** 2021-04-12

**Authors:** Augusto Magalhães, Ana Maria Cunha, Rodrigo Vilares-Morgado, Elisa Leão-Teles, Esmeralda Rodrigues, Manuel Falcão, Ângela Carneiro, Jorge Breda, Fernando Falcão-Reis

**Affiliations:** ^1^Department of Ophthalmology, Centro Hospitalar Universitário de São João, Porto, Portugal; ^2^Pediatric Department, Reference Centre of Inherited Metabolic Diseases, Centro Hospitalar Universitário de São João, Porto, Portugal; ^3^Department of Surgery and Physiology, Faculty of Medicine of University of Porto, Porto, Portugal

## Abstract

**Purpose:**

To describe retinal findings in a patient with mucopolysaccharidosis type I (MPS I) that underwent an early treatment with hematopoietic stem cell transplantation (HSCT) and enzyme replacement therapy (ERT). *Case Report*. We describe a case of a 12-year-old female with a biochemical and genetic diagnosis of MPS I. She underwent HSCT and ERT on the first year of life. The visual acuity was 5/10 in both eyes and she had bilateral grade 2 corneal haze. Spectral domain optical coherence tomography (SD-OCT) revealed thickening of the external limiting membrane (ELM) at the fovea. In the parafoveal and perifoveal regions, SD-OCT displayed a loss of the interdigitation, ellipsoid, and myoid zones and of the ELM accompanied by progressive thinning of the outer nuclear layer. Fundus infrared imaging revealed a hyperreflective ring centred on the fovea and hyporeflective areas in temporal parafoveal regions in both eyes. *En face* OCT imaging revealed two hyperreflective rings on the outer retinal level.

**Conclusion:**

This patient developed macular changes with foveal deposition of hyperreflective material and parafoveal thinning, despite early systemic treatment. Systemic therapies can provide an increase in life expectancy and stabilize visual acuity and corneal clouding, although their effect on retinal degeneration is unknown.

## 1. Introduction

Mucopolysaccharidosis (MPS) are a group of lysosomal storage disorders caused by inborn errors of glycosaminoglycan (GAG) metabolism [1]. MPS type I (MPS I) is an autosomal recessive disease due to deficiency of lysosomal hydrolase *α*-L-iduronidase [2], codified by the IDUA gene at locus 4p16.3 [3]. This enzyme is required to break down heparan and dermatan sulfate. As a result, these metabolites build up in several tissues [1].

The phenotypic spectrum of *α*-L-iduronidase deficiencies includes the mildest form, Scheie syndrome; the intermediate form, Hurler-Scheie syndrome; and the most severe form, Hurler syndrome [2]. Significant multisystemic involvement includes dysostosis multiplex, restrictive and obstructive pulmonary and respiratory disease, valvular disease, ocular and hearing disease, organomegaly, and central and peripheral neurological disease. Progressive neurodegeneration in early childhood characteristically complicates the Hurler phenotype, as opposed to the normal neurological development seen in the Scheie phenotype [1, 4].

Current treatments such as allogeneic hematopoietic stem cell transplantation (HSCT) and enzyme replacement therapy (ERT) have increased the life span of these patients and created the need to improve management of ocular disease [1].

Ocular features of MPS I include corneal clouding, glaucoma, pigmentary retinopathy, optic nerve abnormalities (papilledema and atrophy), ocular motility disorders, and refractive errors [4].

We describe the spectral domain optical coherence tomography (SD-OCT) and infrared and *en face* OCT retinal findings in a 12-year-old patient with MPS I (Hurler's syndrome) who underwent treatment with HSCT and ERT during her first year of life.

## 2. Case Report

A 12-year-old Caucasian female child with the diagnosis of MPS I (Hurler's syndrome), with a severe genotype (homozygous status for the mutation c.1293G>A; p.W402X, exon 9, in the *IDUA* gene) began ERT at 5 months of age and was submitted to HSCT at 12 months. She started a severe graft versus host disease one month later, with important skin and pulmonary and gastrointestinal involvement. She was maintained on ERT for three years after HSCT, with no evidence of antibody development.

The patient presented typical dysmorphic features, short stature, slight cognitive impairment, and also interstitial respiratory disease. Her last analytics revealed a normal alpha-iduronidase leucocyte level (91 nmol/h/mg protein to the normal range: 53–105) and normal urinary GAGs (12 mg/mmol creatinine to the normal range: 4–11), although with an MPS I electrophoretic pattern.

**Figure 1:**
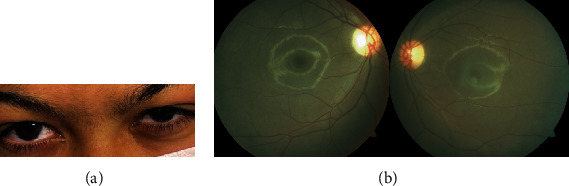
Anterior segment photographs demonstrated mild grade corneal haze bilaterally (a). Fundus photography of the right and left eyes (b).

Ophthalmologically, the best corrected visual acuity (BCVA) was 5/10 in both eyes (hyperopia of 1.5 dioptres). The anterior segment examination demonstrated bilateral grade 2 corneal haze (Figure 1). The corneal pachymetry was 544 *μ*m in the right eye and 562 *μ*m in the left eye, and the anterior chamber depth was 2.70 mm and 2.58 mm, respectively (Pentacam, Oculus, USA).

**Figure 2:**
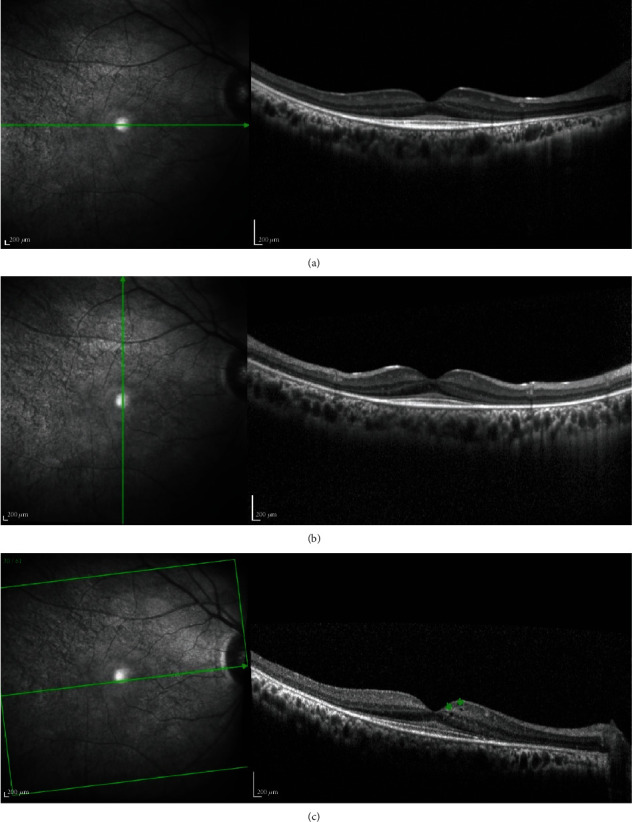
SD-OCT horizontal and vertical scans of fovea in the right eye (a, b). It demonstrates foveal increased thickness of hyperreflective band signal at the ELM, with intact myoid, ellipsoid, and interdigitation zones, RPE, and Bruch's membrane. In the parafoveal and perifoveal region, it demonstrated a progressive loss of interdigitation and ellipsoid zones, myoid zone, and ELM and thinning of the outer nuclear layer. SD-OCT horizontal scan, close to the fovea (c), demonstrated a small cyst in ONL, INL, and GCL (arrows). SD-OCT: spectral domain optical coherence tomography; ELM: external limiting membrane; RPE: retinal pigment epithelium; ONL: outer nuclear layer; INL: inner nuclear layer; GCL: ganglion cell layer.

Fundus examination was clinically normal (Figure 1). However, the SD-OCT assessment revealed in the foveal area, in both eyes, increased thickness of the hyperreflective band of the external limiting membrane (ELM). The other outer retinal bands (the myoid, ellipsoid, and interdigitation zones, the retinal pigment epithelium (RPE), and Bruch's membrane) were within normal limits (Figure 2).

In the parafoveal and perifoveal regions, SD-OCT displayed a loss of the interdigitation, ellipsoid, and myoid zones, loss of the ELM, and thinning and eventual loss of the outer nuclear layer (ONL) (Figure 2). The scans, close to the fovea, also revealed small cysts in the ONL, inner nuclear layer (INL), and ganglion cell layer (GCL) (Figure 2).

**Figure 3:**
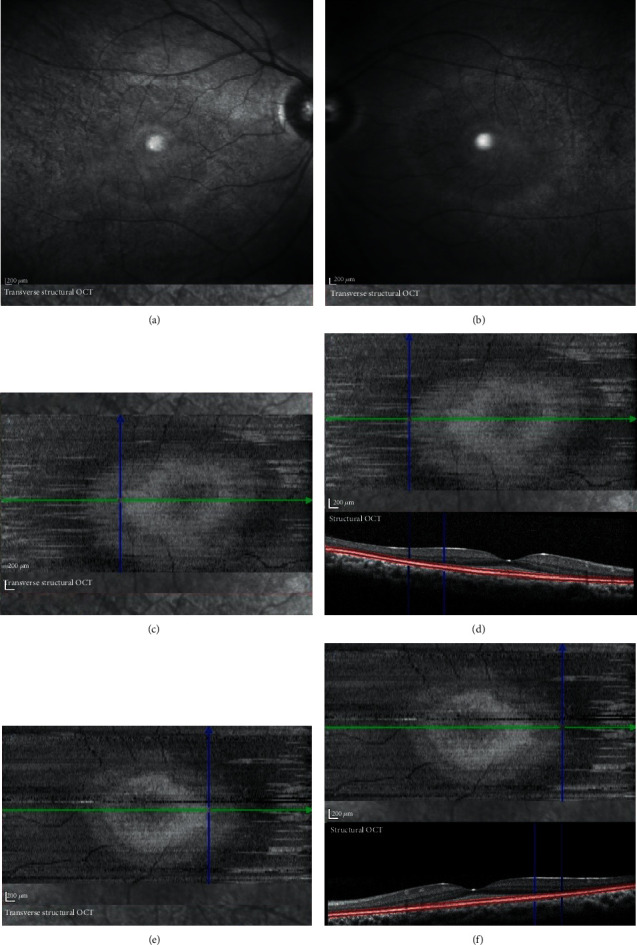
Fundus infrared imaging revealed hyperreflective ring centred in the fovea and hyporeflective areas in temporal parafoveal regions in both eyes (a, b). *En face* SD-OCT images at the level of the outer retina revealed two hyperreflective rings corresponding to the disruption of the ellipsoid zone (c, e) and ELM (d, f). SD-OCT: spectral domain optical coherence tomography; ELM: external limiting membrane.

Fundus infrared imaging revealed a bilateral hyperreflective ring centred on the fovea that was more evident in the left eye. It also revealed hyporeflective areas in the temporal extrafoveal regions in both eyes (Figure 3).

*En face* OCT images at the level of the outer retina revealed two hyperreflective rings. The inner ring corresponded to the limits of the disruption of the ellipsoid zone, and the external ring limits the disruption of the ELM on SD-OCT (Figure 3).

**Figure 4:**
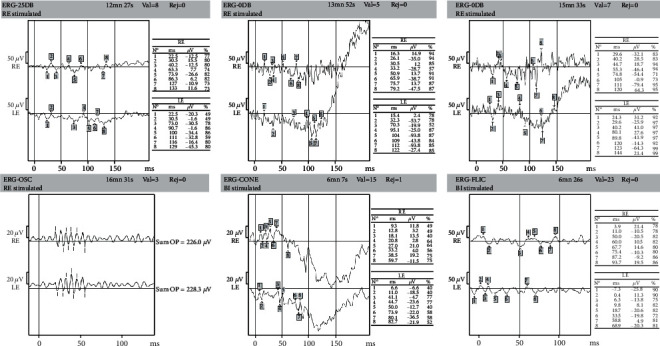
ERG findings. Absence of DA 0.01 ERG response and a DA 10.0 with a diminished a-wave and b-wave. LA 3.0 ERG with a diminished b-wave and absence of LA flicker 30 Hz ERG response. ERG: electroretinography; DA: dark-adapted; LA: light-adapted.

Electroretinography (ERG) was performed according to the International Society for Clinical Electrophysiology of Vision (ISCEV) standards. The ERG findings showed an absence of the dark-adapted (DA) 0.01 ERG response and a DA 10.0 with a diminished a-wave and b-wave. The light-adapted (LA) 3.0 ERG had a diminished b-wave and absence of the LA flicker 30 Hz ERG response (Figure 4).

## 3. Discussion

The main ocular manifestations of MPS I include corneal clouding, glaucoma, retinal pigmentary degeneration, and optic nerve abnormalities [1]. Earlier and more effective treatments have led to an increase in life expectancy of MPS I patients, and so it is currently possible to identify later manifestations of ocular diseases. Moreover, with the advent of new diagnostic technologies, namely, OCT, it is possible to identify retinal structural changes, even in the presence of a clinically normal fundus.

Our SD-OCT findings revealed, in both eyes, an increased thickness of the ELM in the foveal area; a loss of the interdigitation, ellipsoid, and myoid zones, of the ELM; and a thinning of the ONL in parafoveal and perifoveal regions. We also observed small cysts in the ONL, the INL, and the GCL.

Previous studies with SD-OCT in MPS I patients are scarce and include a limited number of patients. The thickening of the ELM at the fovea has been previously described [4–7]. However, in these previous studies, the patients were only treated with ERT, unlike our clinical case that received both HSCT and ERT. At the parafoveal and perifoveal regions, the photoreceptor inner and outer segment thinning has also been reported [5], but not the complete loss of the ellipsoid and myoid zones and the ELM. The thinning of the ONL, as described in our case, is a novel finding. Retinal cysts have been reported, usually at the ONL [4, 5]. Other possible OCT findings, not present in our case, include macular epiretinal membrane and macular edema-like changes [8].

The infrared and *en face* OCT findings correlate and have not been previously described. The fundus infrared image revealed a hyperreflective ring that is evident in the *en face* OCT images at the level of the outer retina, with two hyperreflective rings. These findings correspond to the area of the thickened ELM, and its limits correspond to the ellipsoid zone and ELM disruptions seen on SD-OCT B-scans. The hyperreflective ring has been reported in macular abnormalities such as retinitis pigmentosa [9], but it has not been previously described in MPS patients.

Retinopathy in MPS I occurs due to accumulation of GAG within the RPE cells and the interphotoreceptor matrix, followed by phagocytosis by Muller cells or retinal pigment epithelial cells, leading to progressive loss of photoreceptors [4, 10]. Lazarus et al. suggested that the absence of lysosomal enzymes in the RPE induces RPE hypertrophy and can generate an altered GAG distribution of the interphotoreceptor matrix, which can subsequently affect the photoreceptor's cell-supportive function, leading to photoreceptor degeneration [11].

The cause for the increased hyperreflectance in the region of the ELM observed in our clinical case is not known. It may represent GAG accumulation in Müller cells, undigested abnormal photoreceptor outer segments (although unlikely given that it is internal to the ellipsoid line zone), or abnormal collagen deposition. We propose that our patient has attenuated enzyme action in Müller cells, allowing clearing of degraded GAG. However, at the fovea, the increased number of photoreceptors results in a higher load of GAG overwhelming local enzyme capacity leading to a consequent thickening of the ELM as seen on SD-OCT.

Electrophysiological tests are important for the evaluation of the functional integrity of the retina. In our clinical case, we identified a progressive rod-cone retinal degeneration with an attenuated ERG amplitude, which is the most commonly described pattern [12–14].

As previously reported, HSCT increases life expectancy and provides partial symptomatic improvement and is most effective when initiated early (during the first 2 years of life). Normal bone marrow cells from a matched donor appear to stabilize or improve visual acuity, corneal clouding, and optic nerve swelling in MPS I patients [1, 15]. The ERT replaces the enzyme deficiency via intravenous administration, and when treatment is started early in the patient's life, it is associated with stabilization of corneal clouding. Nevertheless, ERT does not cross the blood-brain barrier and presumably does not cross the blood-retina barrier either [4, 12]. For this reason, there are no reports of improved retinal function. This hypothesis is confirmed by the findings observed in our case report. The patient had a mild corneal clouding but retinal degeneration, despite having undergone early HSCT and being maintained for a long period of time with ERT with normal levels of lysosomal enzyme *α*-L-iduronidase (IDUA).

In conclusion, our patient presented macular changes with foveal deposition of a hyperreflective material at the level of the ELM and parafoveal thinning despite the early systemic treatment with HSCT and ERT and normal alpha-iduronidase levels. Systemic therapies provide an increase in life expectancy and stabilize visual acuity and corneal clouding, despite the fact that they do not seem to modify retinal degeneration. Further studies are required to determine the nature of the material and to truly evaluate the effect and incidence of the current therapies in MPS I retinal changes. With the increase in survival and improved technology, our comprehension of the disease evolution and the effect of available treatment can increase over time.
